# Gigantomastia and Macroprolactinemia Responding to Cabergoline Treatment: A Case Report and Minireview of the Literature

**DOI:** 10.1155/2016/3576024

**Published:** 2016-04-18

**Authors:** Fatma Dilek Dellal, Didem Ozdemir, Cevdet Aydin, Gulfem Kaya, Reyhan Ersoy, Bekir Cakir

**Affiliations:** ^1^Department of Endocrinology and Metabolism, Ataturk Training and Research Hospital, 06800 Ankara, Turkey; ^2^Department of Endocrinology and Metabolism, Faculty of Medicine, Yildirim Beyazit University, 06800 Ankara, Turkey

## Abstract

*Background*. Macroprolactinemia is defined as predominance of high molecular weight prolactin forms in the circulation. Although macroprolactin is considered as a biologically inactive molecule, some authorities suggest treatment in symptomatic cases. Gigantomastia is defined as excess breast tissue and most cases in the literature were treated by surgical intervention.* Case*. A 44-year-old woman was admitted to our clinic with gigantomastia and galactorrhea. The patient had a demand for surgical therapy. In laboratory examination, she had hyperprolactinemia and macroprolactinemia. Pituitary imaging revealed 6 mm microadenoma in right side of the hypophysis. Since she was symptomatic, cabergolin treatment was started. Macroprolactin became negative, breast circumference decreased significantly, and galactorrhea resolved after treatment.* Conclusion*. Gigantomastia might be the presenting symptom in patients with macroprolactinemia. In these patients medical treatment with cabergoline may be used initially as an alternative to surgical approach.

## 1. Introduction

Prolactin (PRL) which is secreted from the pituitary gland is an essential hormone for lactation and has an important role in the development of breast tissue. It regulates lobular and acinar development by binding to cellular receptors on breast tissue and induces production of milk. Hyperprolactinemia secondary to pregnancy or dopamine antagonist treatment causes growth of breast [1]. It was shown that addition of PRL to breast tissue culture stimulates budding structures [2].

Macroprolactinemia is defined as predominance of high molecular weight PRL forms (big-big PRL, MW > 150 kDa) which occurs when PRL monomers form complex with anti-PRL autoantibodies [3]. There are controversies about the clinical importance of macroprolactinemia, the indications for macroprolactin measurement, and the need for treatment in patients with macroprolactinemia. There is not any clinical sign that can help to differentiate macroprolactinemic patients from real hyperprolactinemic ones. Although oligomenorrhea and galactorhea are observed with less frequency in patients with macroprolactinemia, at least one of these symptoms might be seen in many patients [4]. Despite lack of any consensus about routine measurement of macroprolactin, it is recommended to determine its level in patients with asymptomatic hyperprolactinemia [5, 6]. It is thought that macroprolactin is a biologically inactive molecule [7–9]; however some authorities suggest treatment in symptomatic cases [3, 10].

Gigantomastia, macromastia, and breast hypertrophy are terms used as synonym. Although there is no clear definition for gigantomastia, it is generally defined as excess breast tissue. There are various causes of gigantomastia and it can be seen in 10–25% of patients with hyperprolactinemia [11]. Patients present with gigantomastia associated physical and pshycological problems. Physical symptoms and signs are usually related to increased weight of breast tissue such as pain in shoulders, back, neck, and breast. Irritation, erythema, and ulceration under the submammarian fold secondary to hygienic problems might be observed [12]. Surgical interventions, hormonal therapy, or combination of these may be used for treatment. However, it is concluded by many authors that gigantomastia can not be treated medically and surgical intervention is the only option [13]. Here, we report significant clinical improvement with cabergoline treatment in a patient with gigantomastia, macroprolactinemia, and pituitary microadenoma.

## 2. Case Presentation

A 44-year-old women applied to our clinic with enlargement of breast tissue, galactorrhea, and pain in breast and back for 6 months. The patient had normal menstrual cycles and the last pregnancy was 20 years ago. She did not have any history of chronic disease, drug use, or excess weight gain. Family history was negative for breast hypertrophy. The patient had a demand for surgical therapy. In physical examination, weight was 82 kg, height was 162 cm, and body mass index was 31.2 kg/m^2^.

**Figure 1:**
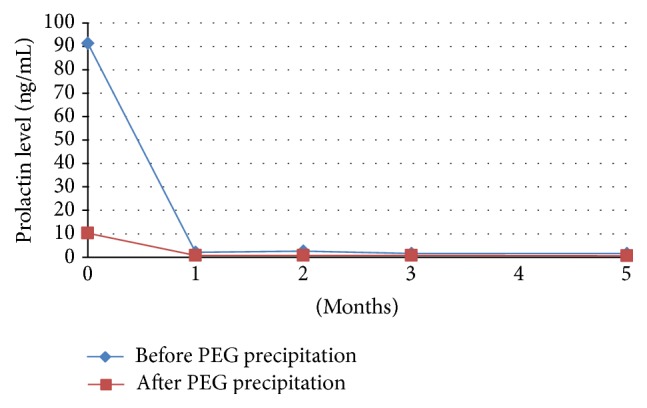
Prolactin levels before and after polyethylene glycol (PEG) precipitation.

**Figure 2:**
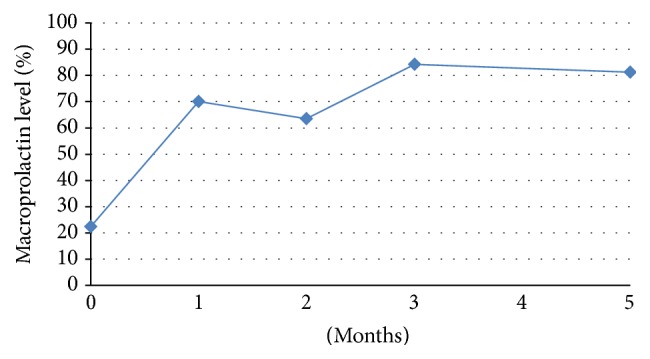
Macroprolactin levels.

Both breasts were hard and there was not any mass by palpation. Breast skin was tense; superficial veins were prominent and dilated. The patient did not have fever and there was not any erythema, warmth, or ulceration on breast skin. Bilateral galactorrhea was observed. The widest horizontal line passing from the areola was determined as the circumference of breast and it was 116 cm. Serum prolactin level was 91.38 ng/mL (15–65 ng/mL) and polyethylene glycol (PEG) preciptation for macroprolactin was positive (22.37%). Prolactin levels before and after PEG precipitation and macroprolactin levels were shown in Figures 1 and 2, respectively. Biochemical analysis and other anterior pituitary hormone levels were normal (Table 1). Basal serum cortisol was 16.07 *μ*g/dL and it was suppressed to 1.4 *μ*g/dL after 1 mg overnight dexamethasone suppression test. Serum was diluted by 1/100 for prolactin measurement to exclude “hook effect” and the result was 87.36 ng/mL. In pituitary magnetic resonance imaging, a nodular lesion suggestive of a 6 mm microadenoma deviating the infindibulum to the left was detected in the right side of the hypophysis. Bilateral breast ultrasonography and mammography were normal.

Since the patient had galactorrhea, cabergoline treatment was started at a dose of 0.5 mg/week. One month after the treatment, macroprolactin was negative and prolactin level was in normal limits. Breast circumference measurements made by the same clinician at the 1st, 2nd, 3rd, and 5th months were 110, 108, 106, and 105 cm, respectively. During follow-up galactorrhea resolved, tenderness recovered, and breast became relaxed. The patient was satisfied with the medical treatment.

## 3. Discussion

The clinical importance of macroprolactinemia is a controversial issue for years. While it is associated with hyperprolactinemic symptoms in some studies [4, 14–16], it is reported not to cause any signs or symptoms in others [8, 11]. This contradiction in the literature may be explained by heterogeneity in the structure of macroprolactin. Anti-PRL antibodies complexed with PRL are in the form of IgG in majority of cases; however antibodies in the form of IgA and IgM were also observed rarely [10].

Macroprolactin synthesis is generally believed to be a peripheral phenomenon. On the other hand, detection of pituitary adenoma in 20% of patients with macroprolactinemia has suggested that its origin might also be the pituitary gland. In addition, it was reported that 20% of patients with macroprolactinemia have galactorrhea and 45% have oligo/amenorrhea [5]. However, coincidental finding of pituitary nonfunctioning adenoma and macroprolactinemia may be a cause of the oligosymptomatic clinical presentation in these patients.

There is no consensus about the definition and classification of gigantomastia and macromastia yet. These two terms are generally used for each other. In general, they are defined as development of excess breast tissue compared to normal population resulting with physical and psychological pathologies. Surgically, enlargement of breast tissue which requires 1500 g reduction per breast may also be described as gigantomastia [17]. In the literature, there are studies reporting different values ranging from 300 to 2000 g for this amount [13]. According to another definition, if the resected breast tissue is under 300 g it is mild, if it is between 300 and 800 g it is moderate, and if it is over 800 gr it is severe macromastia [18]. Gigantomastia is classified etiologically into three main groups: idiopathic (Group 1), imbalance in endogenous hormone production (Group 2), and drug induced (Group 3) group. Group 1 is further devided into two: Group 1a includes obese patients, while Group 1b includes nonobese patients. Gigantomastia cases related to pubertal period and pregnancy are classifed as Group 2a and Group 2b, respectively. Idiopathic gigantomastia has unknown etiology and insidious onset. Suggested mechanisms in Group 2a (pubertal) are increased estrogen receptors in mammary gland and hypersensitivity of receptors to estrogen and progesterone. These patients have sudden disease onset, unilateral or bilateral gigantomastia, and family history. In Group 2b (pregnancy related), possible mechanism of gigantomastia is increased sensitivity to prolactin in mammary gland. Incidence is 1/28000–1/100000 of pregnancies, it has sudden onset and high rate of recurrence. Although possible mechanism in drug induced gigantomastia is unknown, it is commonly associated with other autoimmune diseases [13].

Generally, the first-line treatment of gigantomastia is surgery. Losing weight in Group 1a and withdrawal of medicine in Group 3 are the first treatment steps before surgery. Breast reduction surgery is the most frequently used surgical approach. However, recurrent surgery is required in most cases due to spontaneously continuing breast enlargement or hormonal impulses like pregnancy. In this situation total mastectomy might be an option. Hormonal treatment is generally applied before surgery, but not as a standalone option. Hormonal treatment alternatives present in the literature are bromocriptine, medroxyprogesterone, didrogesterone, tamoxifen, and danazol. Bromocriptine is effective particularly in Group 2b, while tamoxifen, medroxyprogesterone, and didrogesterone are used in Group 2a [13]. There exist no data in the literature about cabergoline treatment for gigantomastia.

In our case, coexistence of macroprolactinemia, gigantomastia, and pituitary adenoma was detected. Macroprolactinemia in the patient may be explained by three possiblities. Firstly, nonfunctioning pituitary adenoma and macroprolactinemia may be just a coincidental finding in this patient. Secondly, pituitary adenoma itself might be the origin of macroprolactin and, lastly, macroprolactin might be synthesized peripherally from breast-derivated PRL. In the literature, there is evidence that supports the first two possibilities. Leslie et al. showed monomeric PRL isoform in pituitary adenoma tissue samples of patients with macroprolactinemia. This result is supportive of the peripheral synthesis of macroprolactin [19]. Conversely, there are two studies demonstrating higher macroprolactin concentrations in adenomatous prolactinoma tissue compared to normal pituitary tissue [20]. Additionally, Lakatos et al. reported 80-year-old male patient with intra- and parasellar pituitary mass and hyperprolactinemia largely in the form of macroprolactin [21]. These cases support the tumoral origin of macroprolactinemia. Although PRL is mainly secreted from the pituitary, studies have shown that bioactive PRL is also synthesized and secreted from adipose and glandular breast tissue [22]. In animal studies, breast-derivated PRL was shown to play an important role particularly in postpartum secretory activity [23]. Breast-derivated PRL production in our patient may also be considered an etiologic factor.

Cabergoline is an ergot derivative which has a selective, prolonged, and dose-dependent inhibitory effect on PRL. Its biological effect is regulated through dopamine, noradrenaline, and serotonin receptors. Stimulation of dopamine-2 receptors in lactotrop cells causes a decline in adenylate cyclase activity by decreasing the intracellular cAMP levels. PRL exerts its proliferative effect on breast tissue via RANKL and IGF-2 [23]. The fact that serum PRL levels are positively correlated with breast density on mammography in postmenopausal women is put forward as an evidence of mitogenic effect of PRL on breast tissue [24]. Cabergoline causes a reduction in cell volume in prolactinoma by early inhibition of secretory mechanisms and late inhibition of gene transcription and PRL synthesis. It also reduces prolactinoma size through perivascular fibrosis and partial cell necrosis [25, 26]. With a similar mechanism, it might cause a decrease in mitogenic activity in breast tissue and consequent reduction in size.

While coexistence of macroprolactinemia and gigantomastia was not reported in the literature previously, Oladele et al. presented 3 cases with hyperprolactinemia and gigantomastia [27]. In the first case, gigantomastia has started in pregnancy and PRL level was minimally increased. The second case was a 25-year-old nulliparous woman with minimally increased PRL levels. These two cases were not treated by dopamine agonists and underwent surgery for gigantomastia. The third patient was a 41-year-old, infertile woman who had a previous history of bromocriptine treatment. Her serum PRL level was high, other pituitary hormone levels were normal, and estradiol and progesterone levels were low. This patient was initially treated with bromocriptine and then a follow-up surgery was performed. PRL levels after surgery were not reported in all three patients. In the literature, other gigantomastia cases treated with cabergoline are all associated with pregnancy. All these patients underwent surgery after bromocriptine treatment [27]. In our case, the recovery of gigantomastia and macroprolactinemia with cabergoline treatment may be suggestive of possible role of macroprolactinemia in the etiology of gigantomastia. Whether the origin of macroprolactinemia was pituitary gland or peripheral tissue could not be identified because there was not any indication for pituitary surgery and histopathological evaluation was not made.

In conclusion, cabergoline seems to be reliable and effective option for the treatment of coexistant gigantomastia and macroprolactinemia, and it can be used as an alternative to surgery. Long term follow-up studies are required to evaluate the possibility of recurrence after withdrawal of cabergoline treatment. Although gigantomastia and macroprolactinemia are generally considered to be benign conditions, pituitary imaging, treatment with a dopamine agonist, and follow-up might be helpful in some cases.

## Figures and Tables

**Table 1:** Biochemical and hormonal profile of the patient.

	Basal	1st month	2nd month	3rd month	5th month
Glucose (mg/dL)	74		79	72	71
Creatinine (mg/dL)	0.74		0.74	0.78	
ALT (U/L)	16	11	11	11	16
TSH (*μ*IU/mL)	1.57	1.53	2.83	3.03	2.85
Free T4 (ng/dL)				1.04	1
Prolactin before PEG precipitation (ng/mL)	**91.38**	2.08	2.58	1.64	1.6
Prolactin after PEG precipitation (ng/mL)	10.22	0.78	0.82	0.69	0.65
Macroprolactin (%)	**22.37**	70	63.57	84.15	81.25
FSH (mIU/mL)	4.04			22.74	
LH (mIU/mL)	4.54			12.69	
Estradiol (pg/mL)	117.8			99.83	
Progesterone (ng/mL)				0.55	
GH (ng/mL)	0.03				
IGF-1 (ng/mL)	115.3				
Cortisol (*μ*g/dL)	16.07				
ACTH (pg/mL)	13.64				
*β*-HCG (mIU/mL)	0.1			0.1	

Glucose (74–106 mg/dL), creatinine (0.5–1.2 mg/dL), ALT (0–33 U/L), TSH (0.27–4.2 *μ*IU/mL), free T4 (0.9–1.7 ng/dL), prolactin (6–29.9 ng/mL), macroprolactin (0–40% positive, 40–60% borderline, and >60% negative), FSH (1.7–21.5 mIU/mL), LH (1–96 mIU/mL), estradiol (12.5–498 pg/mL), progesterone (0.1–3 ng/mL), GH (0–5 ng/mL), IGF-1 (94–252 ng/mL), cortisol (6.2–19.4 *μ*g/dL), ACTH (0–60 pg/mL), and *β*-HCG (0–5 mIU/mL).
